# News and Notes

**Published:** 1908-11

**Authors:** 


### NEWS AND NOTES.

---

New Medical Library.—It is announced that the cost of the new medical building for the Medical and Chirurgical Faculty of Maryland will be $88,000, and of this about $63,000 has already been subscribed.

---

Dr. Charles P. Wertenbaker, of Norfolk, who is attending the convention of military surgeons now in session in this city, was in charge of the United States government health office here during the epidemic of yellow fever in 1905.

---

The College of Physicians and Surgeons of Philadelphia announces that the next award of the AlvarengaPrize, amounting to about $180, will be made July 14, 1909. Essays intended for competition must be received by the secretary of the college by May 1, 1909; each essay must be typewritten; must be without signature; must be plainly marked with a motto; and must be accompanied with a sealed envelope having on its outside the motto of paper, and within the name and address of the author.

---

President Ira Remsen of Johns Hopkins University has resigned the directorship of the chemical laboratory, which he has held since the foundation of the University in 1876.

The reports submitted by the surgeons of the various vessels in the Service, on the subject of organization for battle of the medical department of the United States Navy, shows that there is a lack of uniformity in the organization and in the instruction given to the men regarding their stations and duties, which indicates a need for official action. The wide variation in the types of the vessels in the Service makes the formulation of definite regulations somewhat complicated, but it seems probable that steps will be taken by the Surgeon General to bring about an approximate uniformity of the plan of organization in the establishment of relief and dressing stations on vessels of similar types

---

Colonel George H. Torney, now commanding the Army General Hospital at the Presidio of San Francisco, has been nominated to succeed Brigadier General O'Reily as Surgeon General of the United States Army.

---

Prof. Irving Fisher, political economist of Yale University, who in one of his papers before the recent International Tuberculosis Congress in Washington, declared that consumption costs the people of the United States more than a billion dollars a year, is preparing an exhaustive report for the National Conservation Commission, which will contain not only these figures, but similar data on the economic loss to the country from all other preventable diseases.

---

The authorities of the United States government propose to do much toward educating the public, showing modern appliances and methods of treatment and illustrating the value of sanitary laws and practical hygene at the Alaska-Yukon-Pacific Exposition which will be held in Seattle in 1909. One of the features of the exhibition of the treasury department in the government building will be that of the Public Health and the Marine Hospital Service.

---

The following physicians were noted among those of the Georgia delegation in attendance upon the International Congress on Tuberculosis in Washington, D. C., September 28, October 3rd: Dr. W. E. Adams, Madison; Dr. R. T. Bryant, Turnerville; Dr. M. A. Clark, Macon; Dr. T. D. Coleman, Augusta; Dr. J. M. Crook, Columbus; Dr. J. W. Duncan, Dr. Frank Eskridge, Dr. Marion McH. Hull, and Dr. Louis C. Rughlin, Atlanta; Dr. W. W. Owens, Savannah; and Dr. Henry R. Slack, LaGrange. Most of these visited the hospitals and sanatoria in New York, Philadelphia and Baltimore before returning home.

### REGULAR MEETING FULTON COUNTY MEDICAL SOCIETY.

Carnegie Hall, November 5, at 8 P. M.

Dr Stirling in chair.

Reported by R. R. Daly, M. D.

*Dr. Andrews* exhibited a case of spinal tuberculosis in a young child for the purpose of showing the differentiation of mixed tubercular infection and making the diagnosis in all early cases.

The forearm of the patient is cleansed with soap and water and ether. Then a drop each of the pure bovine, the pure human and the mixed cultures are placed on the skin about two inches apart.

The epidermis is slightly scarified in the center of each drop, and the reaction waited for. In 18 to 24 hours it appears. If the spot of bovine innoculation reacts, the disease is probably of human infection for these two seem antagonistic, and if the human vaccination is excited, the disease is probably bovine.

It was noted that these tests are without any danger and much less annoying than the reactions obtained from instillations into the eyes, or subcutaneous injections.

They are of greatest value in childhood because in adults, particularly those over 40 years of age, the reaction shows some slight tuberculosis and recalls the German idea that if one has lived long enough, he always has some tuberculosis about him.

*Dr. Duncan* discussed the report and confirmed it with his recent experience at the International Conference at Washington, D. C.

*Dr. Calhoun* asked if the test were of use in the negro. He was answered that the reaction showed plainly enough even if the skin were dark.

*Dr. Fischer* reported a case of Abscess of the Lung and operation upon it.

The young man had pneumonia and grip in 1906, and was sent out West for treatment. He was examined then by several different men who confirmed the diagnosis of phthisis. He improved slightly and returned here five months ago.

He showed area of dullness on left side, had irregular fever and what was supposed to be tubercular sputum.

Twenty-five different examinations were made by several competent men and no tubercular germs were found. The sputum seemed different from that of phthisis; it was noticed to flow more freely from him when leaning forward; the cough came usually in the morning after lying long in one position. These led Dr. Fischer to operate. He did so over the heart apex and opened an abscess in the lung which poured out a pint of very foul pus. The patient made a good recovery and felt much better for 30 days. Then pnuemonia in left lung developed. After recovery from this, another area of dullness higher up and nearer the axillary line than the former, was mapped out. The 5th and 6th rib were removed, another foul abscess opened exuding foul pus. The patient recovered and today is well. He was present at the meeting. This case emphasizes the need of careful examination of cases that look so tubercular as to lead one to a preconception they are so. The patient had passed several men who declared him phthisical and indeed his general appearance warranted it. He had passed nearly two years time as a consumptive which might have been spared him. Besides he was in constant danger of death from the unrecognized abscess.

*Dr. Andrews* said that the large abscess cavities suggested a field for the Vaseline-Bismuth-Wax injection.

*Dr. Duncan* related a case of abscess in a negro that was unrecognized till it ruptured.

*Dr. Hodgson's* paper on "Complications of Gonorrhoea in Women" was read by Dr. Ballenger.

Discussed by Dr. Davis who said the subject should be gone over at least once a year if only to keep before the profession the exceeding gravity of the disease. 70 to 80 per cent. of cases in the gynocologic clinic were due to gonorrhoea and at least 40 per cent. in private practice.

The disease is regarded by the public with pernicious levity and they should be made to know that it is difficult to cure.

*Dr. Lokey* suggested that every married woman should be examined for gonorrhoea before partirition to prevent opthalmia neonatorum.

*Dr. Stirling* said women generally should be informed about this disease before marriage in the hope of securing safety. He added that the rheumatism in rheumatic iritis was usually of the gonorrhoeal type.

*Dr. Davis* reported a case of uterus bicornis unicollis.

She began to menstruate at 16, was married at 19. She had been fairly regular but often complained of pain in left side. There was some vaginal distrubance which was treated with ichthyol, etc., and got well. Two months later she missed period and showed pregnancy at 6 1-2 months. She had a premature child that died in 4 days. She convalesced only to return in 3 months. She had menstruated once and missed once. There was bloody flow for 3 or 4 days. She flowed again in a month, always complained of left side. The mass previously felt in left side had increased at least four times and it was thought that this was about to cause an abortion. Upon opening abdomen the true condition was disclosed. The unimpregnated horn was pressed upon the left ovary and adherent to it was causing trouble with the pregnant side. The unimpregnanted horn and that ovary were removed. She made an uneventful recovery and now is doing nicely at the 7th month expecting to go safely to term.

*Dr. Kime* said operations in pregnant women are getting more frequent and removal of tumors dangerous because of their size are successfully removed.

Committees were appointed for the entertainment of the Southern Medical Socity and to investigate the question of caring for all school children.

R. R. DALY.

